# Vertebral Artery Stenosis: A Narrative Review

**DOI:** 10.7759/cureus.28068

**Published:** 2022-08-16

**Authors:** Venkata Sathya Burle, Amelia Panjwani, Kesava Mandalaneni, Sunitha Kollu, Vasavi Rakesh Gorantla

**Affiliations:** 1 Anatomical Sciences, St. George's University School of Medicine, Clarksville, USA; 2 Anatomical Sciences, St. George's University School of Medicine, Whitby, CAN; 3 Neuroscience, St. George's University School of Medicine, St. George's, GRD; 4 Prosthodontics, Mamata Dental College, Khammam, IND; 5 Anatomical Sciences, St. George's University School of Medicine, St. George's, GRD

**Keywords:** neurofibromatosis, fibromuscular dysplasia, calcification, stenosis, vertebral artery

## Abstract

Vertebral artery stenosis (VAS) is the cause of approximately 20% of ischemic strokes in the posterior circulation. There are several causes of vertebral artery stenosis, including atherosclerosis, calcification, dissections, fibromuscular dysplasia, giant cell arteritis, neurofibromatosis type 1, and bony compressions. The most common cause of VAS is atherosclerosis which is derived from the macrophage-induced oxidation of low-density lipoproteins (LDLs), alongside the accumulation of cholesterol. Calcification of the vertebral artery occurs when there is excess calcium and phosphate deposition in the vessel. Dissection of the vertebral artery can lead to the formation of a hematoma causing stenosis of the vertebral artery. Fibromuscular dysplasia can result in stenosis due to the deposition of collagen fibers in the tunica media, intima, or adventitia. Giant cell arteritis, an autoimmune disorder, causes inflammation of the internal elastic membrane resulting in eventual stenosis of the artery. Neurofibromatosis type 1, an autosomal dominant disorder, results in the stenosis of the vertebral artery due to the altered function of neurofibromin. Mechanical compression of the vertebral artery by bone can also cause stenosis of the vertebral artery. Digital subtraction angiography (DSA) is considered the current gold standard in diagnosing vertebral artery stenosis; however, its associated morbidity and mortality have led to increased use of non-invasive techniques such as duplex ultrasonography (DUS), computed tomography angiography (CTA), and magnetic resonance angiography (MRA). Currently, asymptomatic and symptomatic vertebral artery stenoses are treated by risk factor modification and medical treatment. However, it is recommended that surgical (endarterectomy, reconstruction, and decompression) and endovascular (balloon coronary, bare-metal, and drug-eluting stents) treatments are also used for symptomatic vertebral artery stenosis.

## Introduction and background

Vertebral artery stenosis (VAS) is a condition in which the lumen of the vertebral artery is condensed and narrowed. The proximal vertebral artery is the most prevalent location of vertebral artery stenosis [1]. Around 20% of posterior circulation ischemic strokes involve the stenosis of the vertebral artery [2]. The causes of vertebral artery stenosis include arterial calcification, atherosclerotic lesions, dissection lesions, fibromuscular dysplasia, giant cell arteritis, neurofibromatosis, and abnormal bony compression of the vessel [3-5]. Symptomatic vertebral artery stenosis can present with vertigo, vision difficulties, nystagmus, dizziness, loss of consciousness, nausea, and ataxia [6]. If symptomatic VAS is left undiagnosed and unmanaged, it may result in strokes, myocardial infarctions, vertebrobasilar insufficiency (VBI), and sudden death [7]. Diabetes mellitus, hypertension, and hyperlipidemia are common risk factors for vertebral artery stenosis [8-11]. Intracranial vertebral artery stenosis is more common in individuals of Sub-Saharan Africa and East Asia than Caucasians [12]. This article reviews VAS pathophysiology, pathogenesis, diagnostic techniques, risk factors, and management strategies.

## Review

Normal anatomy

Although there are variations in the vertebral artery's origin, course, and branches, this artery has a conventional model [13]. The vertebral artery is typically the first major branch of the subclavian artery on both the left and right sides of the body [14]. It can be divided into four anatomically different segments (V1-V4), where segments V1-V3 are classified as the extracranial vertebral artery, and segment V4 is considered the intracranial vertebral artery [15]. The first segment (V1), known as the proximal or ostial segment, is defined from its origin of branching off the subclavian artery to the sixth cervical vertebrae's transverse foramen [4,16]. The second segment (V2), known as the transverse segment, is defined from the transverse foramina of the sixth cervical vertebrae to the transverse foramina of the second cervical vertebrae [4,16]. The third segment (V3), known as the suboccipital segment, is defined from where the artery leaves the transverse foramina of the second cervical vertebrae to where it pierces the dura mater at the level of the foramen magnum [4,16]. Once the artery pierces the dura mater, it becomes the intracranial vertebral artery [16]. The fourth segment (V4), known as the intracranial segment, is defined from where the artery pierces the dura mater at the foramen magnum to where it joins with the contralateral vertebral artery to form the basilar artery [4,16]. There are two main branches of the V4 segment of the vertebral artery, the anterior spinal artery and the posterior inferior cerebellar artery (PICA) [14]. Additionally, the vertebral artery is one of the providers of blood supply to the circle of Willis, an essential anastomotic structure of the brain [6]. Tortuosity of the vertebral artery is common and is mainly seen in the V1 segment [14]. The tortuous nature and variability of the vertebral artery make it a site for various pathologies and difficulties in identification and diagnosis.

Pathogenesis and pathophysiology

Calcification

Literature indicates that the calcification of vessels plays a critical role in up to 90% of atherosclerotic lesions [17]. Calcification of vasculature occurs when calcium and phosphate deposit in blood vessels [18]. Evidence suggests that vascular calcification is similar to bone remodeling as proteins and hormones related to bone, such as osteocalcin, Runx2, and alkaline phosphatase, have been found in the calcification of vessels, specifically valvular vessels [3]. Vessels are composed of three layers [18]. The innermost layer is the tunica intima, the middle layer is called tunica media, and the outermost layer is called the tunica adventitia [18]. Vascular calcification is classified as either intimal (occurring in the tunica intima) or medial (occurring in the tunica media) [3]. The medial calcification is referred to as a non-occlusive process that results in the hardening of vessels, a decrease in compliance, and an increase in blood pressure [3]. The intimal classification is a subset of vascular calcification that results in atherosclerosis which yields atherosclerotic lesions and stenosis [3]. Therefore, intimal calcification of the vertebral artery can cause vertebral artery stenosis through atherosclerotic lesions.

Atherosclerotic Lesions

The second most common location for plaque buildup in the cerebral vascular system is the vertebral artery [16]. Atherosclerotic lesions are the predominant cause of lesions in the vertebral artery [4]. High cholesterol in the blood triggers atherosclerosis [19]. The increase in cholesterol levels alters the permeability of the tunica intima endothelium [19]. This alteration in permeability enables the adherence of low-density lipoprotein cholesterol (LDL-C) to the tunica intima [19,20]. Following the adherence of LDL-C to the tunica intima, several cytokines like tumor necrosis factor (TNF), interleukin-1β (IL-1β), selectins, and vascular adhesion molecule-1 (VCAM-1) get released from the arterial wall [19,20]. VCAM-1 allows for monocyte adhesion to vessels [19]. A fatty streak is then formed when monocytes mature into foamy macrophages [19]. As a result of monocyte maturation, subendothelial LDL particles begin to oxidize [19]. The oxidized LDLs then promote chemotaxis of scavenger receptors that result in the agglomeration of intracellular cholesterol [19]. The resulting atherosclerotic plaques cause lesions and the stenosis of vessels [19]. Atherosclerotic lesions that yield stenosis have been seen in the extracranial portions of the vertebral artery [4]. Possible complications of extracranial vertebral artery lesions include local thrombosis and ischemia due to a reduction in vertebral artery flow and emboli [4].

Dissection Lesions

Cerebrovascular accidents caused by vertebral artery dissection (VAD) are infrequent in the general population [21]. However, dissection lesions are one of the primary causes of cerebrovascular accidents in populations under 45 years old, with an approximate incidence of 10%-25% [21,22]. Vertebral artery dissections are traumatic, associated with pre-existing arteriopathies, or spontaneous [4]. Spontaneous dissections are associated with the elderly population, whereas traumatic dissections are associated with younger populations [23]. Chiropractic manipulation, spontaneous head movements, cervical trauma, oral contraception, and fibromuscular syndromes like Ehlers-Danlos syndrome type IV and osteogenesis imperfecta type I are examples of dissection lesion sources [4,21-23]. Vertebral artery dissections commonly occur in segment three due to their close association with the atlantoaxial joint, which functions in the rotational movement of the head [4,21]. The process of dissections occurs when the tunica intima tears, blood accumulates, and dissects in the arterial wall [4,21]. As a result of the dissection, thrombosis and hematomas precipitate [4,21]. The hematomas and thrombosis can result in hypoperfusion, stenosis, thromboembolism, Wallenberg syndrome, and strokes [4,21].

Fibromuscular Dysplasia

The third most common cause of lesions in the vertebral artery is fibromuscular dysplasia (FMD) [4]. Fibromuscular dysplasia is a hereditary vascular disease that is non-atherosclerotic and non-inflammatory and causes stenosis, thromboembolism, dissections, fistulas, hypoperfusion of cerebral blood flow, and aneurysms in any network of blood vessels [24,25]. As FMD occurs in any artery, presenting symptoms will vary based on the area of vasculature affected [26]. Fibromuscular dysplasia is classified into medial, intimal, and adventitial [25,26]. Medial FMD is the most common type, and it is characterized by the alternating deposition of collagen in areas of degenerating elastic fibers in the tunica media [25,26]. The deposition of collagen fibers generates areas of alternating dilation and stenosis [25,26]. The second most common form of FMD is intimal, which occurs in the tunica intima of vessels [25,26]. Intimal FMD is characterized by the deposition of collagen in the tunica intima, causing stenosis [25,26]. The final classification of FMD is adventitial FMD [25,26]. It is the least common subtype, and it is classified as the deposition of collagen in the adventitia [25,26]. Adventitial FMD often extends into the connective tissue that surrounds an artery, causing arterial stenosis [25,26].

Unusual Lesion Causing Diseases

In addition to atherosclerotic, dissection, and fibromuscular dysplasia lesions, there are uncommon diseases that also cause lesions [4]. An example of an uncommon disease that can cause bilateral vertebral artery lesions is giant cell arteritis (GCA) [4,27]. GCA is an autoimmune disorder that affects the vasculature by causing inflammation at the internal elastic membrane [27]. The inflammation occurs because of the infiltration of giant cells [27]. Arteries that experience granulomatous inflammation lead to thrombotic obstruction, necrosis, or stenosis [27]. It has been noted that 75%-100% of GCA-affected arteries are vertebral arteries [27]. Neurofibromatosis type 1 (NF-1) is another disease that causes vertebral artery lesions [4,28]. NF-1 is an autosomal dominant disorder that affects tissues like the brain, bone, and blood vessels [28]. Neurofibromin is typically expressed in endothelial and smooth muscle cells of the vasculature [29]. Patients with NF-1 have altered function of neurofibromin which can cause vascular lesions through arterial stenosis, occlusion, aneurysm formation, and arteriovenous fistulas [4,28,29].

Bony Compression

Vertebral arteries may become stenosed due to being compressed at any point along the course of the vertebral artery [5]. The most common anatomical point prone to extracranial compression is at the C1-C2 level due to its association with the atlantoaxial joint and membrane [5]. Causes of vertebral artery stenosis due to compression can be categorized as either primary or acquired causes [5]. Primary causes include muscular hypertrophy of the neck, large osteophytes, fibrous bands, idiopathic skeletal hyperostosis, cervical spondylosis, spondylolisthesis, herniated discs, atlantoaxial hypermobility, hyperflexion, hyperextension, distraction-flexion cervical injuries, and tumors [5,30-35]. Acquired causes include spinal surgery, head trauma, and neck trauma [5].

Compression of the V3 segment of the vertebral artery is most frequently associated with bow hunter’s syndrome [36]. Bow hunter’s syndrome causes vertebrobasilar insufficiency (VBI) by the occlusion or the stenosis of the vertebral artery during head rotation [5]. VBI occurs when the contralateral vertebral artery does not compensate for the reduced blood flow of the compressed vertebral artery [33]. The blood stasis can cause the formation of emboli that gives rise to bow hunter’s stroke in the posterior circulation [36]. Bow hunter’s syndrome typically occurs when the dominant vertebral artery is occluded by head rotation. However, bow hunter’s syndrome was also found in non-dominant vertebral artery occlusions and bilateral vertebral artery occlusions [33].

Clinical manifestations

Stenosis or occlusion of the vertebral artery unilaterally or bilaterally causes decreased artery perfusion and can result in several symptoms of a posterior circulation transient ischemic attack, such as vertigo, ataxia, diplopia, disturbance of speech, and bilateral hemianopia [37,38]. Vertebral artery stenosis can also result in recurring syncope, headaches, recurrent stroke, palsy of cranial nerves, change in consciousness, altered function of the sensory and pyramidal tracts, cerebellar infarcts, and tinnitus [8,24,27,34]. Vertebral artery stenosis can also result in decreased perfusion to the basilar artery and cause several symptoms, including vertigo, dizziness, diplopia, ataxia, dysarthria, nausea, nystagmus, drop attacks, loss of consciousness, motor symptoms, sensory symptoms such as numbness, and an increased risk of experiencing strokes or transient ischemic attacks [6,16,30,32,39]. These symptoms are typically observed when there is stenosis or occlusion of both vertebral arteries [16].

Symptomatic vs. asymptomatic

The true incidence of vertebral artery stenosis is not known due to the cases of asymptomatic stenosis of the vertebral artery. In a study conducted with 3,717 patients, 7.6% (6.8% to 8.5%, CI of 95%) of patients, who exhibited symptoms of atherosclerotic arterial disease, had asymptomatic vertebral artery stenosis or occlusion [40]. Therefore, it was concluded that there is a low risk of posterior circulation stroke in patients with asymptomatic vertebral artery stenosis [40]. The symptoms manifesting in patients with vertebral artery stenosis occur due to various syndromes affecting the vertebral artery. One such syndrome is subclavian steal syndrome (SSS) [18]. In SSS, severe stenosis or occlusion of the subclavian artery, closest to the origin of the vertebral artery, results in the reversal of blood flow in the ipsilateral vertebral artery and shunting of blood from the contralateral vertebral artery into the subclavian artery [14,18]. In addition to SSS, many other causes of stenosis or occlusion of the vertebral artery can result in vertebrobasilar insufficiency (VBI) [41]. VBI occurs due to hypoperfusion of the vertebrobasilar arterial system resulting in vertigo, diplopia, dizziness, loss of consciousness, dysphagia, dysarthria, nausea, ataxia, nystagmus, and numbness [6]. Vertebral artery stenosis is also associated with a high risk of strokes, myocardial infarctions, and sudden death [7].

Diagnostic methods

Vertebral artery stenosis (VAS) accounts for approximately 20% of ischemic attacks and strokes in the posterior circulation [1]. The symptomatic presentation of vertebral artery stenosis can vary and can present with vertigo, diplopia, dizziness, nystagmus, nausea, ataxia, and numbness [6]. If vertebral artery stenosis is left undiagnosed and untreated, there is a high risk of cerebrovascular accidents, myocardial infarctions, and sudden death, thus, making it imperative to evaluate the relevant techniques in diagnosing VAS [7].

The vertebral artery anatomy is complex, tortuous, and variable, with intracranial and extracranial segments [14]; therefore, making it difficult to diagnose and identify despite advances in imaging techniques over time. There are invasive and non-invasive imaging techniques. Duplex ultrasonography is a non-invasive imaging strategy used as the standard initial screening of patients suspected of having VAS [16]. It is a safe and cost-effective investigation method [18,20]. However, DUS does not accurately depict the degree of vascular stenosis, and the vertebral artery origin cannot be imaged in every patient [18,20,42]. Although the employment of color Doppler ultrasonography (color-DUS) and Doppler parameters such as the ratio of peak systolic velocity (PSVr) has enhanced the efficacy of VAS visualization, meta-analyses exemplify the lower sensitivity of color-DUS compared to other non-invasive techniques like CTA and MRA [15]. In vascular syndromes like subclavian steal syndrome (SSS), DUS is an appropriate screening tool [43]. However, MRA and CTA techniques are employed to receive a confirmatory diagnosis due to their accuracy [43]. Digital subtraction angiography (DSA), also referred to as intra-arterial angiography (IAA), is an invasive diagnostic technique that is considered to be the most accurate in diagnosing VAS [1,12]. Although DSA is considered the most precise technique to diagnose VAS, it is the riskiest, most invasive, expensive, and time-consuming technique [44].

Digital Subtraction Angiography

Digital subtraction angiography (DSA), also known as intra-arterial angiography (IAA), is an invasive procedure regarded as the gold standard imaging technique for diagnosing vertebral artery stenosis, disease, and VBI [1,12,14,16,44]. Before revascularization in a patient with symptomatic posterior cerebral ischemia, DSA is preferred over CTA and MRA because they do not reliably outline the origin of the vertebral artery [19]. Although DSA has low morbidity and associated mortality, it is the most invasive, expensive, risky, and time-consuming diagnostic technique available for vertebral artery stenosis [1,16,32,44]. DSA has been associated with transient neurological events, permanent neurological deficits, adverse reactions to contrast mediums, groin hematoma, stroke, carotid, vertebral, femoral, and iliac artery dissections [32]. There is a 1.5% chance of neurological complications, and recent studies suggest that this chance of neurological complications is even higher [32]. Various studies have concluded that non-invasive imaging techniques such as CTA, contrast-enhanced magnetic resonance angiography (CE-MRA), and sonography are safer than DSA; therefore, they are replacing DSA as a reliable and safe diagnostic testing for stenosis and disease of the vertebral artery [12,45].

Duplex Ultrasound and Color Doppler Sonography

Duplex ultrasonography (DUS) is the most common and standardized test for the initial screening and diagnosis of vertebral artery stenosis (VAS) and subclavian steal syndrome (SSS) as it is a safe, accurate, non-invasive, and cost-effective diagnostic method [1,6,18,20]. The intricate location, tortuosity, small diameter, and perpendicular position of the origin of the vertebral artery make it challenging to detect on ultrasound and can only be imaged in 60% of subjects [1,42]. To combat this disadvantage, the employment of color flow imaging in Doppler ultrasound and the establishment of Doppler parameters have increased the effectiveness of Doppler imaging [42]. A study conducted in Turkey recognized PSVr as the most effective Doppler parameter to be used alongside color Doppler sonography when diagnosing vertebral artery stenosis [42]. However, an Austrian study stated that color Doppler sonography was still not effective at visualizing all the segments of every vertebral artery [46]. A meta-analysis highlighted that the sensitivity and specificity of color-DUS were 70.2% and 97.7%, respectively [15]. The same meta-analysis also noted that DUS had a significantly lower sensitivity rate than CTA (100%) and CE-MRA (93.3%) [15]. Moreover, DUS does not accurately demonstrate the degree of stenosis in arteries [18,20].

Computed Tomography Angiography and Magnetic Resonance Angiography

Computed tomography angiography (CTA) is a non-invasive imaging tool to evaluate vertebral artery stenosis and disease [12]. CTA is superior to DSA because it can image the extracranial portion of the vertebral artery while avoiding the potential complications of using catheter angiography [1]. One study concluded that CTA could distinguish between a kinked vertebral artery and a stenosed vertebral artery due to atherosclerotic plaques [1]. However, CTA is also associated with challenges and disadvantages. Studies show that the CTA imaging technique underestimates the prevalence and degree of stenosis in the ostial vertebral artery [47]. Additionally, CTA has difficulties in accurately identifying the diameter of the lumen of extensively calcified arteries [37]. CTA is not the best imaging technique for patients who have a history of renal disease due to the high proton intensity and radiation that it exposes patients to [15,18].

Magnetic resonance angiography (MRA), similar to CTA, is also a non-invasive imaging technique used to evaluate vertebral artery stenosis and disease [12]. Imaging severe stenosis of the vertebral artery, such as the ostial portion, poses a challenge for MRA [37]. MRA overestimates the stenosis of the ostial vertebral artery; however, the use of contrast mediums resolves this challenge [1,47]. Contrast-enhanced MRAs (CE-MRAs), compared to catheter angiography, yield higher-resolution images of all extracranial cervical vessels [2]. Additionally, a sensitivity of 100% and specificity of 85% for the accuracy between conventional angiography and CE-MRA of greater than 50% stenosis of a vertebral artery occlusion in patients who experienced an acute ischemic stroke were found [48]. Therefore, indicating that CE-MRA can be used as a better alternative to detect vertebral artery stenosis [48], CE-MRA also has higher sensitivities and specificities for stenosis of the extracranial vertebral artery than MRA without contrast or DUS [15]. CE-MRA, however, cannot be used with patients who have pacemakers or other metallic devices [15].

Although CTA and MRA are not able to accurately highlight the origin of the vertebral artery, both imaging techniques are associated with higher sensitivities than DUS [12,15,32,39,47]. The sensitivities and specificities of CTA and CE-MRA are 100% and 95.2% and 93.9% and 94.8%, respectively, while DUS’s sensitivity is 70% [15,32]. Therefore, CTA and CE-MRA are currently viewed as more promising imaging techniques than DUS to evaluate vertebral artery stenosis.

Risk factor modification

Similar to several other conditions, the first line of treatment for the disease of the vertebral artery is the modification of risk factors, including physical activity, maintaining a healthy diet, and smoking cessation [12]. Cigarette smoking has been indicated as a risk factor in various causes of vertebral artery stenosis, such as vertebral artery hypoplasia, V1 diseases, and vertebral artery dissections; therefore, smoking cessation may help reduce the incidence of vertebral artery stenosis [9,10,49]. Studies show conflicting data pertaining to whether increased age has a direct correlation with the development of atherosclerosis and calcification of the vertebral artery [50,51]. Additionally, several other risk factors and comorbidities such as hypertension, diabetes mellitus, coronary artery disease, hyperlipidemia, hyperhomocysteinemia, alcohol consumption, obesity in men, and peripheral vascular disease have been associated with causes of vertebral artery stenosis [8-11,52]. Management of these risk factors and comorbidities may help decrease the incidence of vertebral artery stenosis caused by V1 lesions, vertebral artery dissections, vertebrobasilar stenosis, and vertebrobasilar calcification [8-11,52].

Current management strategies

Medical Management

Medical management is utilized in patients with either symptomatic or asymptomatic vertebral artery stenosis [12]. One of the various causes of stenosis of the vertebral artery is atherosclerosis; therefore, medical treatment of its risk factors (hypertension, hyperlipidemia, and diabetes mellitus) is mainly seen [53]. Antihypertensive treatment is recommended to treat hypertension by lowering blood pressure below 140/90 mmHg [12,53]. Statins with or without LDL-lowering therapy and bile acid sequestrants or niacin are used to treat hyperlipidemia by lowering LDL cholesterol below 100 mg/dL [12,47,53]. Physical activity, glucose-lowering medications, dieting, and a statin-type lipid-lowering medication are used to treat diabetes mellitus by lowering glucose levels and reducing LDL levels to around or below 70 mg/dL [53]. It is recommended that patients diagnosed with vertebral artery stenosis due to atherosclerosis or mechanical compression of the vertebral artery be placed on antiplatelet medications such as aspirin, aspirin with modified-release dipyridamole, clopidogrel, or ticlopidine to prevent strokes, myocardial infarctions, and transient ischemic attacks [12,47,53]. In the Warfarin-Aspirin Symptomatic Intracranial Disease (WASID), it was concluded that warfarin might be more effective in preventing strokes for patients with symptomatic vertebral artery stenosis [7].

Surgical Treatment

Surgical revascularization of the vertebral artery is done via endarterectomy or reconstruction surgery [1,16]. Endarterectomy is the removal of atherosclerotic stenosis in the vertebral artery and is a complex procedure with poor success rates due to the intricate location of and access to the vertebral artery [1]. Complications of endarterectomy of the vertebral artery include vocal cord paresis, lymphoceles, fistulas, and lung collapse [1]. Reconstruction surgery of the vertebral artery is a technique that involves the dissection and the transposition of the vertebral artery to either the subclavian, thyrocervical trunk arteries, common carotid, or internal carotid artery [1,54]. In one study, the combined stroke and mortality rate of proximal extracranial vertebral artery reconstruction was less than 2%, and 92% of patients experienced patency 10 years post-operation [1]. The same study found that distal extracranial vertebral artery reconstruction was less successful with a 6% combined stroke and mortality rate, and 80% of patients experienced patency five years post-operation [1]. A study of 55 patients who underwent reconstruction surgery due to VBI caused by stenosis or occlusion of the vertebral artery indicated patency in most patients with various postoperative complications [54]. The complications experienced by patients included syncope, dizziness, temporary vocal cord paresis, elevated hemidiaphragm, and temporary brachial plexus weakness [54]. Moreover, out of the 54.5% of patients who exhibited transient Horner's syndrome, 13.3% of the patient's transient Horner's syndrome became permanent [54]. Although surgical treatment of VAS has shown some success, it is an invasive and complicated procedure associated with substantial postoperative complications and mortality [1,54]. The ideal treatment for anatomical compression of the vertebral artery by bony structures is surgical resection of the compressing structure, decompression of the vertebral artery, or endovascular treatment [5,35,36,55,56].

Endovascular Treatment

Surgical revascularization of symptomatic vertebral artery stenosis is performed less frequently due to the growing use of endovascular techniques such as percutaneous transluminal angioplasty (PTA) and stenting [1]. Angioplasty with stenting is a minimally invasive and highly successful endovascular procedure that dilates stenosed arteries [57]. Typically, the stenosed arteries are infiltrated by atherosclerotic plaques [57]. Endovascular stenting of the vertebral artery (VA) is conducted using the transfemoral or the transradial route [20,58]. The type of stent used can have a significant impact on the outcome of the procedure. In a study conducted in Germany of 38 patients with flexible balloon coronary stents to treat VAS, no new cerebrovascular accidents occurred, and vertebrobasilar ischemia was prevented [59]. However, using flexible balloon coronary stents resulted in a 36% restenosis rate in patients [59]. It is presumed in the literature that the high restenosis rates of the vertebral artery when using coronary stents are attributed to the fact that coronary stents lack the properties to restrain the bending and recoil forces of the vertebral artery [60]. To counteract the high stenosis rates, physicians have explored, and the literature has referenced, the use of bare-metal stents (BMS) and drug-eluting stents (DES) in vertebral artery stenosis. In a Polish study of 392 patients, the success rates and periprocedural complication rates for DES and BMS were 96.7% and 1.4% and 94.6% and 2.2%, respectively [58]. Additionally, the biolimus DES stent has a restenosis rate of 12.9%, and the stainless steel BMS stent had a restenosis rate of 17.8% [58]. The success, complication, and restenosis rates of BMS and DES are similar, with DES being slightly more effective. However, the literature demonstrates that BMS and DES are significantly more effective than coronary stents because of their lower restenosis rates [58,60].

Studies indicate that endovascular treatment of symptomatic vertebral artery stenosis is a safe and successful procedure [38,58,61,62]. In a study of 105 patients in the United States, every patient with symptomatic extracranial vertebral artery stenosis achieved technical success (100%), and 90.5% of patients had clinical success [61]. Furthermore, a study on 73 symptomatic patients in 2014 by Radak et al. highlighted that no deaths occurred during the endovascular procedure and while recovering in the hospital [38]. Moreover, the study also indicated that during the follow-up period, which ranged from two to 144 months (mean: 44.3 + 31.2 months), there was a 10.3% restenosis rate [38]. Moreover, long-term treatment success rates were 98.4%, 87.3%, and 87.3% after one, three, and seven years, respectively [38]. Another study comparing basic medical treatment (BMT) to stenting showed a 60% lower long-term risk for fatal and non-fatal strokes in patients with stents than in patients who received basic medical treatment [63]. In another study conducted on outcomes of endovascular treatment, 98.8% of patients achieved technical success [64]. Thus, stenting in symptomatic vertebral stenosis is considered a safe, durable, and effective form of managing vertebral artery stenosis with a relatively low risk of complication [1,32,37,38,59,61,63]. Risk factors and diagnostic methods are summarized in Table 1.

**Table 1:** Risk factors, clinical manifestation, diagnostic methods, and management techniques of vertebral artery stenosis DE: drug-eluting stent, BMS: bare-metal stent, ISR: in-stent restenosis, ISO: in-stent occlusion, VBI: vertebrobasilar insufficiency, WASID: Warfarin-Aspirin Symptomatic Intracranial Disease, MRA: magnetic resonance angiography, CE-MRA: contrast-enhanced magnetic resonance angiography, CTA: computed tomography angiography, DUS: duplex ultrasonography, PSVr: ratio of peak systolic velocity, LDL: low-density lipoprotein, VA: vertebral artery, RVAO: rotational vertebral artery occlusion, CAD: coronary artery disease, VBAC: vertebrobasilar artery calcification, BMT: basic medical treatment.

	Author	Country	Study Population	Findings	Conclusion
1	Markus et al., 2017 [63]	United Kingdom	182 patients	The patients whose vertebral arteries were stented showed approximately a 60% lower risk for fatal and non-fatal strokes when compared to the patients who received basic medical treatment.	The long-term risk of a fatal or non-fatal stroke of a stenosed vertebral artery treated through stenting is less when compared to BMT (basic medical treatment) after 3.5 years.
2	Jenkins et al., 2010 [61]	United States	105 patients	All patients with symptomatic vertebral artery stenosis (100%) had technical success, which is defined as no in-hospital stroke or death despite potential residual stenosis of 30% or less after the endovascular stenting procedure. About 90.5% of patients achieved clinical success, which is defined as the resolution of vertebrobasilar system symptoms.	Endovascular stenting as a treatment for symptomatic vertebral artery stenosis demonstrated a high success rate.
3	Antoniou et al., 2012 [62]	Meta-analysis	42 studies	Endovascular treatment included angioplasty, stenting, or both and resulted in a success rate of 97%, 1.5% transient ischemic attack, 1.1% stroke and death, 8% recurring symptoms of vertebrobasilar insufficiency, 23% restenosis, and 9% reintervention.	Treatment of vertebral artery stenosis utilizing endovascular treatment indicates a high success rate and a low risk of adverse side effects; however, it has been associated with notable restenosis and reintervention rates.
4	Weber et al., 2005 [59]	Germany	38 patients	The use of flexible balloon coronary stents prevented vertebrobasilar ischemia, resulted in no new strokes, and resulted in approximately 38% restenosis in all of the patients in this study who had symptomatic stenosis of the proximal vertebral artery.	Using flexible balloon coronary stents to treat proximal vertebral artery stenosis was demonstrated to prevent vertebrobasilar ischemia and its related symptoms successfully; however, it failed to successfully prevent restenosis of the vertebral artery.
5	Katada et al., 1983 [50]	Japan	3,648 patients	This study concluded that 3.4% of scans revealed calcifications in either one or both of the vertebral arteries. There was not a significant difference in calcification incidence between males and females. There was no calcification of the vertebral artery for patients under the age of 40, but the incidence of the calcification started and increased from the fifth decade onward.	Although there was no correlation between vertebral artery calcification and sex, there was a strong correlation between increasing age and the incidence of vertebral artery calcification.
6	Solberg et al., 1971 [51]	Oslo, Norway, Guatemala	961 patients	The incidence of atherosclerosis in the vertebral artery did not differ based on the patient's geographical location, age, and sex.	The incidence of vertebral artery atherosclerosis must be influenced by factors other than the patient's residence, age, and sex.
7	Maciejewski et al., 2019 [58]	Poland	392 patients	The success rates for DES and BMS were 96.7% and 94.6% (p=0.103), respectively. The periprocedural complication rates for DES and BMS were 1.4% and 2.2% (p=0.565), respectively. The ISR/ISO rates for DES and BMS are 22.8% and 19.4% (p=0.635), respectively. The stainless steel stent had the lowest in-stent restenosis rate of 17.8% for BMS, while the biolimus stent had the lowest in-stent restenosis rate of 12.9% for DES.	The rate of success, complication, and ISR/ISO rates of DES and BMS are very similar, indicating that both have similar effectiveness for treating vertebral artery stenosis.
8	No authors, 1998 [7]	United States	68 patients	The WASID study group concluded that compared to aspirin, warfarin demonstrated a lower ischemic stroke rate and was associated with a greater rate of complications related to hemorrhage in both symptomatic intracranial vertebral artery and basilar stenosis. However, this difference was concluded to not be statistically significant.	Compared to aspirin, warfarin could be a better drug for preventing strokes in patients with intracranial vertebral artery and basilar stenosis; however, further investigations must occur.
9	Lima Neto et al., 2017 [39]	Meta-analysis	28 studies	Arteriographs are an important diagnostic technique for VBI but are invasive. MRA is used widely to locate stenoses or occluded neck and intracranial arteries. Transcranial Doppler is a non-invasive and cheap diagnostic test that detects blood flow in the major intracranial arteries. The sensibility and specificity of MRA are 93.9% and 94.8%, of angiotomography are 100% and 95.2%, and of transcranial Doppler are 70.2% and 97.7%, respectively.	Although arteriographs are important diagnostic tools to detect VBI, due to their invasive nature, tests such as MRA and transcranial Doppler may be better options to utilize when wanting to detect VBI.
10	Louw et al., 1990 [31]	South Africa	12 patients	In this study, it was found that 76% of cases for closed vertebral artery occlusion were due to distraction-flexion cervical injuries with a facet joint dislocation, while 7% of the cases were due to hyperextension cervical injuries accompanied either with or without rotation and lateral flexion.	Of the non-penetrating injuries to the extracranial vertebral artery, distraction-flexion injuries with a joint dislocation of the facet are more common than hyperextension injuries.
11	Kim et al., 2013 [48]	Korea	774 patients	Conventional angiography is an invasive procedure, whereas CE-MRA is a non-invasive procedure. There was a sensitivity of 100% and specificity of 85% for the accuracy between conventional angiography and CE-MRA of greater than 50% stenosis at a vertebral artery occlusion. The concordance rate between conventional angiography and CE-MRA was 83.7% for patients who experienced an acute ischemic stroke.	CE-MRA may be a better alternative for evaluating vertebral artery occlusion in patients who have experienced an acute ischemic stroke.
12	Khan et al., 2007 [15]	Meta-analysis	11 studies	The sensitivities and specificities of CTA are 100% and 95.2%, of CE-MRA are 93.9% and 94.8%, and of color duplex are 70.2% and 97.7%, respectively. This study also concluded that DUS is associated with lower sensitivity than CTA and CE-MRA.	Both CE-MRA and CTA diagnostic tests might have higher sensitivity rates when compared to DUS for the ability to diagnose vertebral artery stenosis; therefore, they might be better to use.
13	Kim et al., 2005 [1]	Korea	935 patients	The prevalence of stenosis in the proximal vertebral artery and the distal vertebral/basilar artery in the study population was 12.9% and 5.5%, in the asymptomatic group was 3.3% and 0.5%, in the minor symptom group was 8.3% and 2.1%, in the cardiac group was 13.3% and 6.7%, in the hemorrhagic group was 19.2% and 7,7%, in the anterior circulation infarct group was 27.3% and 8.3%, and in the posterior circulation infarct group was 44.4% and 36.1%, respectively.	Stenosis of the vertebral artery was more prevalent in the proximal part of the artery than in the distal part.
14	Yurdakul and Tola, 2011 [42]	Turkey	48 patients	This study recognized PSVr as the best Doppler parameter in diagnosing vertebral artery stenosis because the artery's tortuosity, minuscule diameter, location, and perpendicular position to the subclavian artery make it difficult to diagnose with color Doppler sonography alone.	Diagnosing proximal vertebral arteries are complex; therefore, to accurately diagnose with color Doppler sonography, PSVr is the best parameter to consider.
15	Amarenco et al., 1990 [24]	France	56 patients	Among the 56 patients involved in the cerebellar infarct study, vertebral artery occlusions made up 76% of infarction.	In this study, the majority of cerebellar infarcts involved vertebral artery occlusions.
16	Feng et al., 2020 [8]	China	343 patients	Patients with vertebrobasilar artery stenosis (n=100) had a higher incidence of hypertension (74%), diabetes mellitus (33%), and hyperhomocysteinemia (27%) in comparison to the symmetric (n=74), asymmetric (n=127), and hypoplastic (n=42) vertebrobasilar artery patients.	Hypertension, diabetes mellitus, and hyperhomocysteinemia are linked to vertebrobasilar artery stenosis.
17	Dinç et al., 2021 [49]	Turkey	609 patients	This study demonstrated that being male (p=0.01) and smoking (p=0.008) are associated with vertebral artery hypoplasia. Age, hypertension, and LDL levels showed no significant association with vertebral artery hypoplasia.	Being male and smoking were common risk factors in patients with vertebral artery hypoplasia. Age, hypertension, and LDL did not have a significant association with vertebral artery hypoplasia.
18	Wityk et al., 1998 [9]	United States	407 patients	Among the 80 individuals who had a lesion in V1 of the vertebral artery, cigarette smoking (N =50), hypertension (N=74), and coronary artery disease (N=48) were the most common risk factors.	Cigarette smoking, hypertension, and CAD are associated with V1 lesions of the vertebral artery.
19	Radak et al., 2014 [38]	Belgrade, Serbia, Switzerland	73 patients	The endovascular treatment procedure to treat vertebral artery stenosis was successful in 68 out of the 73 patients. No deaths occurred during the operation and while recovering in the hospital. The follow-up period ranged from two to 144 months (mean: 44.3 + 31.2 months), and only seven responses were detected. The endovascular treatment success rates were 98.4%, 87.3%, and 87.3% after one, three, and seven years, respectively.	Endovascular treatment of vertebral artery stenosis is a safe and successful procedure.
20	Rüegg et al., 2003 [27]	United States	Three patients	Giant cell arteritis was found to cause bilateral vertebral artery occlusion in the extracranial portions of the vertebral artery in three patients.	Giant cell arteritis may be a cause of vertebral artery stenosis.
21	Trattnig et al., 1990 [46]	Austria	42 patients	When using color Doppler sonography, in 47.6% of patients, every branch of both vertebral arteries was seen; in 19.1% of patients, only one section of both vertebral arteries was not seen, and in 33.3% of patients, more than one section of the vertebral artery was unable to be visualized.	Color Doppler sonography is not effective in seeing all segments of the vertebral arteries.
22	Compter et al., 2011 [40]	Netherlands	3,717 patients	The study demonstrated that 7.6% (95% CI, 6.8% to 8.5%) of the subjects with atherosclerosis had asymptomatic VA stenosis.	Atherosclerosis has a link to asymptomatic VA stenosis.
23	Choi et al., 2013 [34]	South Korea	21 patients	Every patient with RVAO had vertigo. In addition, vertigo was accompanied by tinnitus (38%), syncope (24%), or refractive errors (19%).	RVAO is associated with vertigo, tinnitus, syncope, and refractive errors.
24	Hsu et al., 2013 [10]	Taiwan	17 patients	Smoking was seen in four of 17 patients, hypertension was noted in three of 17 patients, alcohol consumption was observed in two of 17 patients, and hyperlipidemia in two of 17 patients.	Smoking, hypertension, alcohol consumption, and hyperlipidemia are common risk factors in vertebral artery dissections.
25	Moufarrij et al., 1984 [11]	United States	96 patients	The following conditions were found to be associated with vertebral artery stenosis: CAD (64%), hypertension (53%), peripheral vascular disease (52%), and diabetes mellitus (19%).	CAD, hypertension, peripheral vascular disease, and diabetes mellitus are associated conditions in vertebral artery stenosis.
26	van der Toorn et al., 2019 [52]	Netherlands	2,483 patients	Obesity was highlighted to be a risk factor for VBAC in men. However, in women, there was an inverse relationship between VBAC and obesity. This phenomenon is potentially due to the role of estrogen in fat mass.	Obesity is a risk factor for VBAC in men and not in women.

## Conclusions

Vertebral artery stenosis can be challenging to identify, diagnose, and treat due to its variability and tortuous nature. There are several causes of vertebral artery stenosis. Intimal calcification of vascular tissue is an occlusive process that leads to atherosclerotic lesions and stenosis. Atherosclerotic lesions are generated by the oxidation of LDLs and the agglomeration of intracellular cholesterol. The consequent atherosclerotic plaques yield stenotic lesions of the vertebral artery. Dissection lesions occur when the tunica intima of a blood vessel tears, and blood accumulates (dissects) in the arterial wall. The dissection leads to stenosis, thrombosis, and hematomas. Vertebral arteries may also become stenosed due to compression by bony structures. Other uncommon causes of VAS are fibromuscular syndromes, neurofibromatosis, and autoimmune disorders like giant cell arteritis. Several diagnostic techniques can evaluate VAS, including DSA, DUS, CTA, and MRA. DSA is considered the gold standard for diagnosing vertebral artery stenosis. However, due to the invasive nature and associated complications of DSA, non-invasive diagnostic techniques such as duplex ultrasonography (DUS), computed tomography angiography (CTA), and magnetic resonance angiography (MRA) have taken precedence. Although DUS is an appropriate, cost-effective, and non-invasive screening tool, MRA and CTA techniques are often used to confirm the diagnosis. The course of action for the treatment of VAS will vary based on whether the patient is symptomatic or asymptomatic. The initial treatment for VAS is the modification of risk factors and the use of medical treatments such as antihypertensive, antiplatelet, glucose-lowering, and statin medications. Surgical and endovascular interventions are recommended only for symptomatic patients. Surgical intervention involves either endarterectomy, reconstruction, or surgical decompression of the vertebrae. Endovascular stenting using balloon coronary stents, bare-metal stents (BMS), and drug-eluting stents (DES) has been used to treat VAS; however, the literature recommends using DES over both BMS and balloon coronary stents due to restenosis rates.

## References

[1] Kim SH, Lee JS, Kwon OK, Han MK, Kim JH (2005). Prevalence study of proximal vertebral artery stenosis using high-resolution contrast-enhanced magnetic resonance angiography. Acta Radiol.

[2] Cloud GC, Markus HS (2003). Diagnosis and management of vertebral artery stenosis. QJM.

[3] Wu M, Rementer C, Giachelli CM (2013). Vascular calcification: an update on mechanisms and challenges in treatment. Calcif Tissue Int.

[4] George B, Laurian C (1987). Anatomy. The Vertebral Artery: Pathology and Surgery.

[5] Montano M, Alman K, Smith MJ, Boghosian G, Enochs WS (2021). Bow hunter's syndrome: a rare cause of vertebrobasilar insufficiency. Radiol Case Rep.

[6] Pollard H, Rigby S, Moritz G, Lau C (1998). Subclavian steal syndrome: a review. Australas Chiropr Osteopathy.

[7] (1998). Prognosis of patients with symptomatic vertebral or basilar artery stenosis. The Warfarin-Aspirin Symptomatic Intracranial Disease (WASID) Study Group. Stroke.

[8] Feng Y, Liu J, Fan T (2020). Vertebral artery stenoses contribute to the development of diffuse plaques in the basilar artery. Front Bioeng Biotechnol.

[9] Wityk RJ, Chang HM, Rosengart A, Han WC, DeWitt LD, Pessin MS, Caplan LR (1998). Proximal extracranial vertebral artery disease in the New England Medical Center Posterior Circulation Registry. Arch Neurol.

[10] Hsu CY, Cheng CY, Lee JD (2013). Clinical features and outcomes of spinal cord infarction following vertebral artery dissection: a systematic review of the literature. Neurol Res.

[11] Moufarrij NA, Little JR, Furlan AJ, Williams G, Marzewski DJ (1984). Vertebral artery stenosis: long-term follow-up. Stroke.

[12] Naylor AR, Ricco JB, de Borst GJ (2018). Editor's choice: management of atherosclerotic carotid and vertebral artery disease: 2017 Clinical Practice Guidelines of the European Society for Vascular Surgery (ESVS). Eur J Vasc Endovasc Surg.

[13] Magklara EP, Pantelia ET, Solia E (2021). Vertebral artery variations revised: origin, course, branches and embryonic development. Folia Morphol (Warsz).

[14] Tay KY, U-King-Im JM, Trivedi RA (2005). Imaging the vertebral artery. Eur Radiol.

[15] Khan S, Cloud GC, Kerry S, Markus HS (2007). Imaging of vertebral artery stenosis: a systematic review. J Neurol Neurosurg Psychiatry.

[16] Madonis SM, Jenkins JS (2021). Vertebral artery stenosis. Prog Cardiovasc Dis.

[17] Wu XH, Chen XY, Wang LJ, Wong KS (2016). Intracranial artery calcification and its clinical significance. J Clin Neurol.

[18] Ahmed MA, Parwani D, Mahawar A, Gorantla VR (2022). Subclavian artery calcification: a narrative review. Cureus.

[19] Bergheanu SC, Bodde MC, Jukema JW (2017). Pathophysiology and treatment of atherosclerosis: current view and future perspective on lipoprotein modification treatment. Neth Heart J.

[20] Ahmed M, McPherson R, Abruzzo A, Thomas SE, Gorantla VR (2021). Carotid artery calcification: what we know so far. Cureus.

[21] Britt T, Agarwal S (2022). Vertebral artery dissection. StatPearls [Internet].

[22] Flis CM, Jäger HR, Sidhu PS (2007). Carotid and vertebral artery dissections: clinical aspects, imaging features and endovascular treatment. Eur Radiol.

[23] Hutting N, Verhagen AP, Vijverman V, Keesenberg MD, Dixon G, Scholten-Peeters GG (2013). Diagnostic accuracy of premanipulative vertebrobasilar insufficiency tests: a systematic review. Man Ther.

[24] Amarenco P, Hauw JJ, Gautier JC (1990). Arterial pathology in cerebellar infarction. Stroke.

[25] Olin JW, Gornik HL, Bacharach JM (2014). Fibromuscular dysplasia: state of the science and critical unanswered questions: a scientific statement from the American Heart Association. Circulation.

[26] Baradhi KM, Bream P (2022). Fibromuscular dysplasia. StatPearls [Internet].

[27] Rüegg S, Engelter S, Jeanneret C, Hetzel A, Probst A, Steck AJ, Lyrer P (2003). Bilateral vertebral artery occlusion resulting from giant cell arteritis: report of 3 cases and review of the literature. Medicine.

[28] Pereira VM, Geiprasert S, Krings T (2007). Extracranial vertebral artery involvement in neurofibromatosis type I: report of four cases and literature review. Interv Neuroradiol.

[29] Hamilton SJ, Friedman JM (2000). Insights into the pathogenesis of neurofibromatosis 1 vasculopathy. Clin Genet.

[30] Piñol I, Ramirez M, Saló G, Ros AM, Blanch AL (2013). Symptomatic vertebral artery stenosis secondary to cervical spondylolisthesis. Spine (Phila Pa 1976).

[31] Louw JA, Mafoyane NA, Small B, Neser CP (1990). Occlusion of the vertebral artery in cervical spine dislocations. J Bone Joint Surg Br.

[32] Kocak B, Korkmazer B, Islak C, Kocer N, Kizilkilic O (2012). Endovascular treatment of extracranial vertebral artery stenosis. World J Radiol.

[33] George B, Bruneau M, Spetzler RF Extrinsic compression bow hunter’s stroke. Pathology and Surgery Around the Vertebral Artery.

[34] Choi KD, Choi JH, Kim JS (2013). Rotational vertebral artery occlusion: mechanisms and long-term outcome. Stroke.

[35] Fleming JB, Vora TK, Harrigan MR (2013). Rare case of bilateral vertebral artery stenosis caused by C4-5 spondylotic changes manifesting with bilateral bow hunter's syndrome. World Neurosurg.

[36] Cornelius JF, Pop R, Fricia M, George B, Chibbaro S (2019). Compression syndromes of the vertebral artery at the craniocervical junction. Acta Neurochir Suppl.

[37] Jenkins JS, Stewart M (2017). Endovascular treatment of vertebral artery stenosis. Prog Cardiovasc Dis.

[38] Radak D, Babic S, Sagic D, Tanaskovic S, Kovacevic V, Otasevic P, Rancic Z (2014). Endovascular treatment of symptomatic high-grade vertebral artery stenosis. J Vasc Surg.

[39] Lima Neto AC, Bittar R, Gattas GS, Bor-Seng-Shu E, Oliveira ML, Monsanto RD, Bittar LF (2017). Pathophysiology and diagnosis of vertebrobasilar insufficiency: a review of the literature. Int Arch Otorhinolaryngol.

[40] Compter A, van der Worp HB, Algra A, Kappelle LJ (2011). Prevalence and prognosis of asymptomatic vertebral artery origin stenosis in patients with clinically manifest arterial disease. Stroke.

[41] Psillas G, Kekes G, Constantinidis J, Triaridis S, Vital V (2007). Subclavian steal syndrome: neurotological manifestations. Acta Otorhinolaryngol Ital.

[42] Yurdakul M, Tola M (2011). Doppler criteria for identifying proximal vertebral artery stenosis of 50% or more. J Ultrasound Med.

[43] Osiro S, Zurada A, Gielecki J, Shoja MM, Tubbs RS, Loukas M (2012). A review of subclavian steal syndrome with clinical correlation. Med Sci Monit.

[44] Nguyen-Huynh MN, Wintermark M, English J, Lam J, Vittinghoff E, Smith WS, Johnston SC (2008). How accurate is CT angiography in evaluating intracranial atherosclerotic disease?. Stroke.

[45] Hua Y, Meng XF, Jia LY, Ling C, Miao ZR, Ling F, Liu JB (2009). Color Doppler imaging evaluation of proximal vertebral artery stenosis. AJR Am J Roentgenol.

[46] Trattnig S, Hübsch P, Schuster H, Pölzleitner D (1990). Color-coded Doppler imaging of normal vertebral arteries. Stroke.

[47] Aboyans V, Ricco JB, Bartelink ME (2018). Editor's choice: 2017 ESC guidelines on the diagnosis and treatment of peripheral arterial diseases, in collaboration with the European Society for Vascular Surgery (ESVS). Eur J Vasc Endovasc Surg.

[48] Kim YJ, Lee JH, Choi JW, Roh HG, Chun YI, Lee JS, Kim HY (2013). Long-term outcome of vertebral artery origin stenosis in patients with acute ischemic stroke. BMC Neurol.

[49] Dinç Y, Özpar R, Emir B, Hakyemez B, Bakar M (2021). Vertebral artery hypoplasia as an independent risk factor of posterior circulation atherosclerosis and ischemic stroke. Medicine (Baltimore).

[50] Katada K, Kanno T, Sano H, Shinomiya Y, Koga S (1983). Calcification of the vertebral artery. AJNR Am J Neuroradiol.

[51] Solberg LA, Eggen DA (1971). Localization and sequence of development of atherosclerotic lesions in the carotid and vertebral arteries. Circulation.

[52] van der Toorn JE, Engelkes SR, Ikram MK, Ikram MA, Vernooij MW, Kavousi M, Bos D (2019). Vertebrobasilar artery calcification: prevalence and risk factors in the general population. Atherosclerosis.

[53] Brott TG, Halperin JL, Abbara S (2011). 2011 ASA/ACCF/AHA/AANN/AANS/ACR/ASNR/CNS/SAIP/SCAI/SIR/SNIS/SVM/SVS guideline on the management of patients with extracranial carotid and vertebral artery disease: executive summary. A report of the American College of Cardiology Foundation/American Heart Association Task Force on Practice Guidelines, and the American Stroke Association, American Association of Neuroscience Nurses, American Association of Neurological Surgeons, American College of Radiology, American Society of Neuroradiology, Congress of Neurological Surgeons, Society of Atherosclerosis Imaging and Prevention, Society for Cardiovascular Angiography and Interventions, Society of Interventional Radiology, Society of NeuroInterventional Surgery, Society for Vascular Medicine, and Society for Vascular Surgery. Circulation.

[54] Diaz FG, Ausman JI, de los Reyes RA, Pearce J, Shrontz C, Pak H, Turcotte J (1984). Surgical reconstruction of the proximal vertebral artery. J Neurosurg.

[55] Cornelius JF, George B, N'dri Oka D, Spiriev T, Steiger HJ, Hänggi D (2012). Bow-hunter's syndrome caused by dynamic vertebral artery stenosis at the cranio-cervical junction: a management algorithm based on a systematic review and a clinical series. Neurosurg Rev.

[56] Judy BF, Theodore N (2021). Bow hunter's syndrome. World Neurosurg.

[57] Majeed H, Chowdhury YS (2022). Percutaneous transluminal angioplasty and balloon catheters. StatPearls [Internet].

[58] Maciejewski DR, Pieniazek P, Tekieli L (2019). Comparison of drug-eluting and bare metal stents for extracranial vertebral artery stenting. Postepy Kardiol Interwencyjnej.

[59] Weber W, Mayer TE, Henkes H (2005). Efficacy of stent angioplasty for symptomatic stenoses of the proximal vertebral artery. Eur J Radiol.

[60] Werner M, Bräunlich S, Ulrich M (2010). Drug-eluting stents for the treatment of vertebral artery origin stenosis. J Endovasc Ther.

[61] Jenkins JS, Patel SN, White CJ (2010). Endovascular stenting for vertebral artery stenosis. J Am Coll Cardiol.

[62] Antoniou GA, Murray D, Georgiadis GS (2012). Percutaneous transluminal angioplasty and stenting in patients with proximal vertebral artery stenosis. J Vasc Surg.

[63] Markus HS, Larsson SC, Kuker W, Schulz UG, Ford I, Rothwell PM, Clifton A (2017). Stenting for symptomatic vertebral artery stenosis: The Vertebral Artery Ischaemia Stenting Trial. Neurology.

[64] Borhani Haghighi A, Edgell RC, Cruz-Flores S, Zaidat OO (2011). Vertebral artery origin stenosis and its treatment. J Stroke Cerebrovasc Dis.

